# Evaluating inflammatory activity in Crohn’s disease by cross-sectional imaging techniques

**DOI:** 10.1590/0100-3984.2018.0096

**Published:** 2020

**Authors:** Bruno Cunha Fialho Cantarelli, Rafael Santiago de Oliveira, Aldo Maurici Araújo Alves, Bruno Jucá Ribeiro, Fernanda Velloni, Giuseppe D’Ippolito

**Affiliations:** 1 Escola Paulista de Medicina da Universidade Federal de São Paulo (EPM-Unifesp), São Paulo, SP, Brazil.; 2 Diagnósticos da América S/A, Barueri, SP, Brazil.

**Keywords:** Crohn disease, Magnetic resonance imaging, Tomography, X-ray computed, Inflammatory bowel diseases, Intestine, small

## Abstract

The evaluation of inflammatory bowel activity in patients with Crohn’s disease has traditionally been a challenge, mainly because of the difficulty in gaining endoscopic access to the small bowel. Historically, barium-based contrast studies were the only option for the evaluation of inflammatory activity in Crohn’s disease. They were gradually replaced by cross-sectional imaging techniques, computed tomography enterography (CTE) and magnetic resonance enterography (MRE) now being the modalities of choice for such evaluations. Those two imaging methods have provided important information regarding intestinal wall involvement and extra-intestinal manifestations of Crohn’s disease, not only assessing lesion characteristics and complications but also quantifying inflammatory bowel activity. The objective of this article is to review the main technical aspects of CTE and MRE, together with their indications, contraindications, and limitations, as well as the CTE and MRE imaging characteristics of inflammatory activity in Crohn’s disease.

## INTRODUCTION

Crohn’s disease is a chronic granulomatous inflammatory disorder of unknown etiology, characterized by transmural inflammation of the gastrointestinal tract. Its diagnosis results from the analysis of clinical data (collected through anamnesis, complete physical examination, and complete proctological examination) and data from endoscopic, radiological, and histological studies, as well as from laboratory tests^1^, cross-sectional imaging methods, such as computed tomography (CT) and magnetic resonance imaging (MRI), being more and more widely used^2^. Barium contrast studies (e.g., barium swallow and barium enema), traditionally used in the diagnosis and management of patients with Crohn’s disease, have lost ground to cross-sectional methods, which are able to evaluate not only the transmural involvement of the bowel but also the common extraintestinal manifestations and complications associated with the disease (for example, there is a recognized relationship between inflammatory intestinal diseases and primary sclerosing cholangitis). In addition, these methods have proved useful in the evaluation of the inflammatory activity of the disease, as long as specific protocols such as computed tomography enterography (CTE) and magnetic resonance enterography (MRE) are followed for acquisition of images.

It is known that Crohn’s disease is marked by periods of remission and relapse, and it is important to monitor the intensity of inflammation, in order to assess the effectiveness of the treatment, rule out complications, and prevent its progression^3^. Although endoscopy has been considered the gold-standard method for the evaluation of inflammatory activity, it has the major limitation of not allowing the routine evaluation of the whole intestine or the diagnosis of some of the main complications of the disease-obstacles which are overcome by cross-sectional imaging methods.

The objective of this article was to review the indications, examination techniques, advantages, disadvantages, and imaging characteristics of CTE and MRE in the evaluation of inflammatory activity in Crohn’s disease.

## CTE

CTE consists of a CT scan of the abdomen with a protocol specific for evaluating the small bowel. It differs from conventional abdominal CT in three aspects: it should be obtained with thin slices, which are conducive to multiplanar reconstructions with high spatial resolution; the images are obtained in the enteric phase of contrast enhancement (45-60 s after intravenous injection of contrast medium); and distension of the small bowel loops is required for a proper evaluation of their walls. Therefore, neutral oral contrast media, such as water, milk, mannitol, sorbitol, and methylcellulose are used, as are, preferably, solutions with polyethylene glycol, due to their efficiency and good acceptance by patients^4,5^. The intestinal distension is considered satisfactory when a caliber greater than 2 cm is obtained in the small bowel^5,6^.

Whereas neutral contrast media can identify the intestinal mucosa with greater precision^4^, positive oral contrast media hinder the assessment of the thickness and enhancement of the intestinal wall, and should therefore not be used. Exceptions are made when fistulas or perforations are suspected, because any extravasation is more easily identified with the use of positive oral contrast media^5,6^.

The volume of oral contrast varies from 1500 mL to 2000 mL, being administered in a rhythmic and constant manner for 45-60 min before the examination. Prokinetic agents can be used in order to ensure an adequate flow of contrast in the small bowel loops and to achieve adequate distension. The intravenous use of iodinated contrast medium is crucial to assess the state of the intestinal mucosa and to characterize the complications inherent to Crohn’s disease; it is administered by injection pump at a flow rate of 3-4 mL/s and a volume of 100-150 mL. Generally, only one phase of the examination (the enteric phase of contrast enhancement) is obtained, eliminating the phases without intravenous contrast and the delayed phases. This strategy has proved efficient and efficacious, allowing the dose of radiation to be kept at levels considered safe and acceptable-below 10 mSv^7-9^.

Because patients with Crohn’s disease are usually diagnosed at an early age and require long-term follow-up with imaging examinations, concern over radiation is a crucial factor. Desmond et al.^10^ found that the use of CT accounted for 77.2% of the diagnostic radiation exposure in patients with Crohn’s disease, the cumulative effective dose being found to be high (> 75 mSv) in over 15% of the patients evaluated. This is why the physician and the radiologist should both be familiar with the strategies available to minimize the radiation dose. Lower voltage and amperage, which can reduce the doses of radiation without impeding the diagnostic performance of the examination, can be achieved through methods such as automatic dose modulation^11^. Another strategy, based on the use of reconstruction algorithms that use iterative approaches, already available in many models of equipment from virtually all manufacturers, maintaining the image quality and reducing the dose by 35-72%^7,8,11^, allows the examination to be made with an exposure of less than 2 mSv.

As shown in Table 1, CTE has the following advantages over MRE: it is more widely available; it takes less time and is more affordable; radiologists are generally more familiar with it; it is less susceptible to motion artifacts (including peristalsis); it has better spatial resolution; there is usually no need for sedation; and it is safe for patients with pacemakers or metallic implants. However, it also has some disadvantages, mainly that it uses ionizing radiation, produces lower contrast between the structures, and requires the use of contrast media with a higher risk of nephrotoxicity^12^.

**Table 1:** Summary of the main advantages and disadvantages of CTE and MRE in the evaluation of patients with Crohn's disease.

Variable	CTE	MRE
Ionizing radiation	Present	Absent
Duration of examination	Short (< 5 min)	Long (30-40 min)
Spatial resolution	Higher	Lower
Contrast among structures	Lower	Higher
Availability	Widely available	Only at large centers
Cost	Lower	Higher
Intravenous contrast	Iodinated, greater nephrotoxicity	Gadolinium, lower nephrotoxicity
Motion artifacts	Low susceptibility	High susceptibility
Dynamic functional image acquisition	Restricted by ionizing radiation	Possible

Choosing between CTE and MRE for patients with Crohn’s disease can generate uncertainty for attending physicians. In a recent study, Bruining et al.^13^ listed the potential factors that influence this choice and identified those that favor CTE: patient age over 35 years; suspicion of sepsis or intra-abdominal collections that may require intervention; a first enterography in a patient with acute symptoms; other causes of diarrhea having been excluded; and any contraindication to MRI. However, MRE should be considered for patients who have previously undergone CTE; for patients ≤ 35 years of age; for pregnant women; for the evaluation of treatment responses; for patients in non-acute phases of the disease; for patients under suspicion of having a perianal fistula; and for patients who are allergic to iodinated contrast. 

## MRE

For the evaluation of the small bowel, MRI has, until recently, been relegated to a secondary role, mainly due to long image acquisition times and frequent motion artifacts, from peristalsis and from breathing. However, technological advances have made its use more robust, especially after the development of more efficient coils and pulse sequences with short acquisition times and high spatial and temporal resolution^2^.

**Figure 1:**
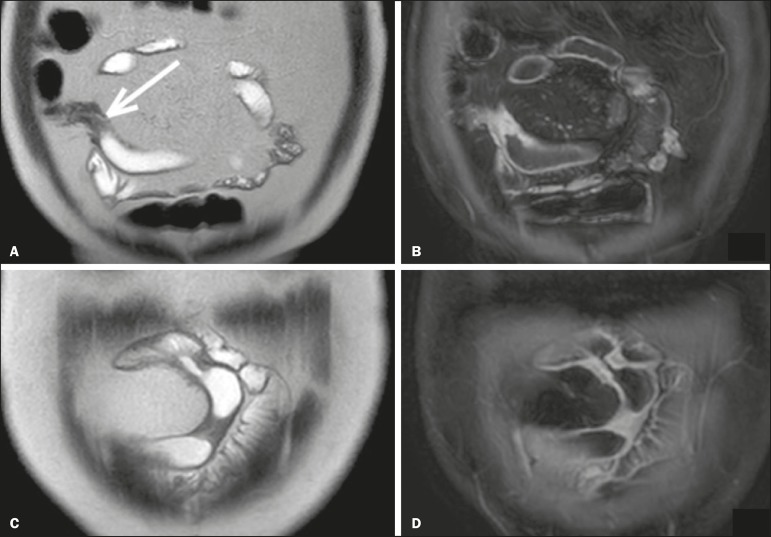
Coronal T2-weighted MRE sequence (**A,B**) and coronal, paramagnetic contrast-enhanced, fat-saturated T1-weighted MRE sequence (**C,D**) showing signs of fibrosis and mild stenosis (arrow) in the distal ileum (**A,C**) and inflammatory activity in the medium ileum (**B,D**). Also note the use of the biphasic oral contrast (polyethylene glycol) distending the intestinal lumen, with high signal intensity in the T2-weighted sequence and low signal intensity in the T1-weighted sequence.

In MRE, the need for distension of the bowel loops for a proper assessment of small bowel diseases is the same as that inherent to CTE, with the administration of contrast agents either orally or via nasojejunal tube (referred to as enteroclysis). Although the distension achieved with enteroclysis is better than that achieved with oral contrast administration, the latter is preferable because of its greater availability, lower complexity, lower cost, and better acceptance by patients, as well as because several studies have shown no significant difference between CTE and MRE in the detection of active inflammatory disease^9,14^. The contrast agents used in MRE are classified according to the signal properties in T1- and T2-weighted sequences. Negative (superparamagnetic) contrast agents have low signal intensity in T1- and T2-weighted sequences, and positive contrast agents (such as vegetable oil) have high signal intensity in both, whereas the signal intensity varies depending on the weighting when biphasic contrast agents are used. The most commonly used biphasic agents are those that produce low signal intensity on T1-weighted images and optimize the evaluation of the enhancement of the intestinal mucosa after their intravenous injection and high signal intensity on T2-weighted images, which allows better evaluation of the anatomy and of the progression of enteric contrast agents, including sugars (sorbitol or mannitol), water, and polyethylene glycol, the last being the most widely used (Figure 1).

The use of intravenous paramagnetic contrast medium in MRE is a routine method for the detection of areas of mucosal hyperenhancement, which is indicative of inflammatory activity in Crohn’s disease. One suggested protocol is to inject 0.2 mmol/kg of paramagnetic contrast at approximately 3 mL/s and to start the image acquisition 45 s afterward. The oral contrast is administered in a manner similar to that described for CTE. Because ionizing radiation is not used in MRE, it is possible to acquire multiple contrast-enhanced images without concern for the deleterious effects of radiation, allowing the dynamic evaluation of the contrast enhancement, distensibility, and motility of the bowel loops^15^. However, the continuous, cumulative exposure of these patients to the paramagnetic contrast medium is a cause for concern, especially in pediatric patients who must undergo MRE multiple times over the course of the disease and during follow-up. Although the consequences of retention and accumulation of gadolinium in the various tissues of the body are not well established^16^, protocols without the use of paramagnetic contrast medium are becoming more and more common. One recent study assessed the impact of paramagnetic contrast medium on the evaluation of Crohn’s disease by MRE in pediatric patients, showing that it is not necessary for the assessment of inflammatory activity in the small bowel, although it is important for the assessment of penetrating perianal disease^17^.

Antispasmodic agents, for reducing peristalsis and consequently motion artifacts, are more useful in MRE than in CTE, glucagon and butylscopolamine^2^ being the most widely used. the latter and to administer it intravenously at the beginning of the examination. 

An MRE examination requires high-speed T1- and T2-weighted sequences, preferably acquired during a single breath hold. Diffusion-weighted sequences are also acquired, because the findings thus obtained can correlate with the inflammatory activity of Crohn’s disease, b values of 50 s/mm^2^ and 900 s/mm^2^ being used at our facility. The assessment of bowel loop motility provides more information than does conventional, static MRI and can be achieved by dynamic acquisition, popularly known as kinematic MRI, that uses T2-weighted gradient echo sequences^18^.

As summarized in Table 1, MRE offers multiple advantages over CTE^15,19^: it does not use ionizing radiation, which is beneficial for patients requiring long-term imaging follow-up and makes it applicable in pregnant women (preferably without the use of intravenous contrast medium); it provides greater contrast among the abdominal structures; it allows the acquisition of functional images in real time, facilitating the differentiation between physiological contraction and established stenosis; and it allows the use of paramagnetic contrast in patients who are allergic to iodine. Another advantage of MRE over CTE is its superiority in the evaluation of anorectal inflammatory disease, which allows better characterization of the extent of the disease in the anal canal^20^. When perianal disease is suspected, MRI studies directed toward the region provide accurate information about the relationship between fistulas and the sphincter complex, the structures of the pelvic floor, and the levator ani muscle. Such studies include the acquisition of high-resolution multiplanar sequences aligned with the axis of the anal canal, allowing proper classification of the type of fistula and consequent better preoperative evaluation^21^.

There are also some recognized limitations of MRE in comparison with CTE^15,19^: limited availability; higher cost; longer examination time; lower spatial and temporal resolution; greater susceptibility to motion artifacts; and a smaller number of radiologists with experience in its interpretation. The last item is relevant, given that examiner experience has been found to have no substantial impact on the reproducibility of the assessment of patients with Crohn’s disease when CTE is used^22^.

## RADIOLOGICAL CRITERIA FOR INFLAMMATORY ACTIVITY

A number of classification systems have been developed in order to differentiate among the various phenotypes of Crohn’s disease, which have distinct clinical manifestations. Among them is the Montreal Classification of Inflammatory Bowel Disease, which differentiates the phenotypes on the basis of the presence or absence of stenotic or penetrating disease, which often coexist, and whether there is an accompanying perianal fistula^23^.

**Figure 2:**
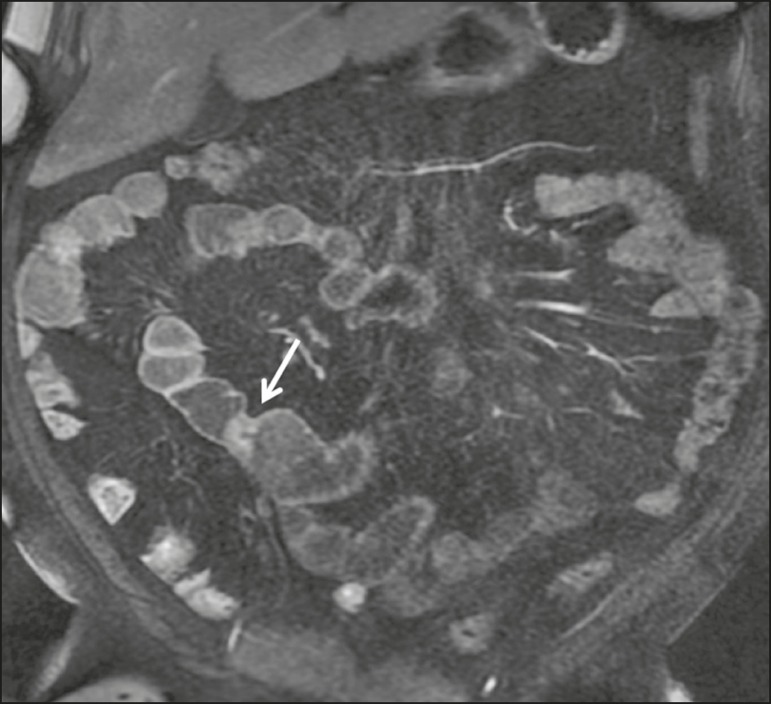
Coronal, paramagnetic contrast-enhanced (gadolinium), fat saturated T1-weighted MRE sequence showing wall thickening with focal stenosis (arrow) and dilatation of the prestenotic segment.

**Figure 3:**
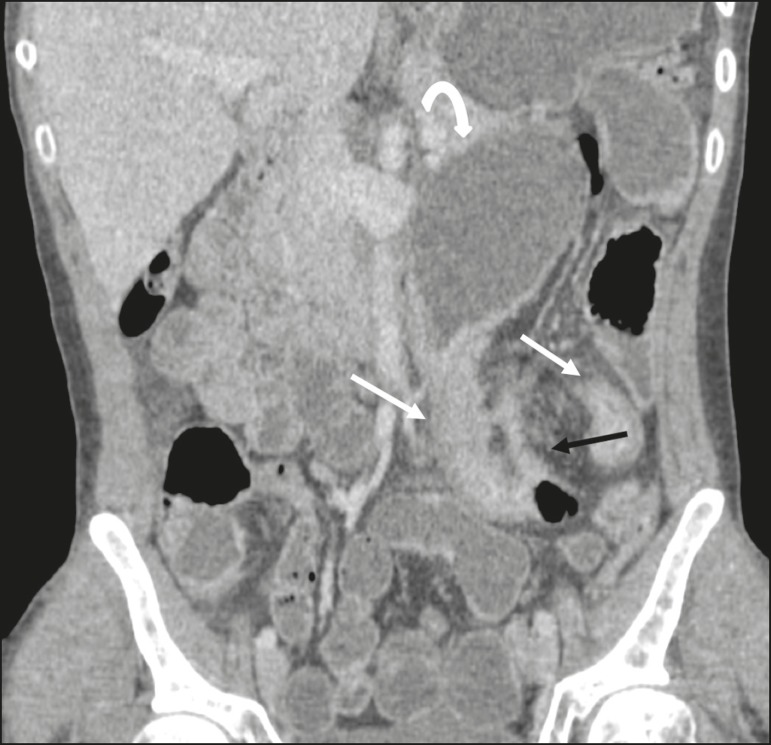
Coronal, contrast-enhanced CTE image showing stenotic, asymmetric wall thickening (straight white arrows) with prestenotic dilatation (curved white arrow). Note also the jejuno-jejunal fistulous tract, indicating penetrating disease, partially characterized in the image (black arrow).

**Figure 4:**
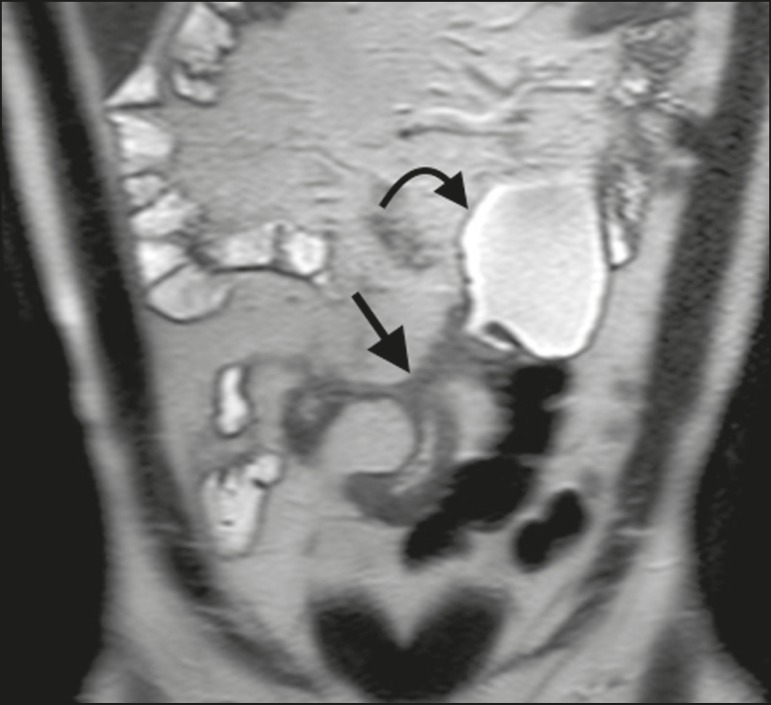
Coronal T2-weighted MRE sequence showing stenosis and enteroenteric fistula in the distal ileum, with the “clover-like” sign (straight arrow). Note also the upstream dilatation of the prestenotic segment (curved arrow).

**Figure 5:**
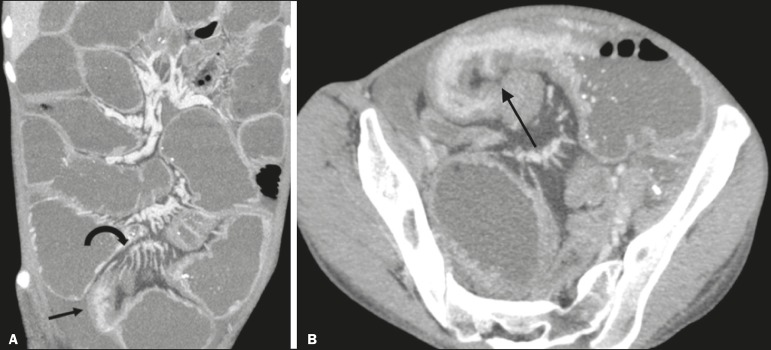
**A:** CTE image with coronal reconstruction, after intravenous and oral contrast, showing stenotic wall thickening with mucosal hyperenhancement (straight arrow) in the distal ileum, resulting in obstruction with consequent diffuse dilatation of upstream loops. There is also engorgement of the mesenteric vessels, known as the “comb sign” (curved arrow). **B:** Axial image of the same patient, showing a long stretch of stenotic thickening in the distal ileum (arrow).

**Figure 6:**
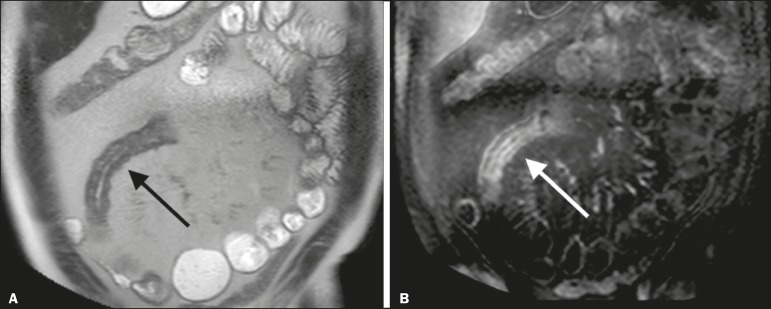
Coronal T2-weighted MRE sequence (**A**) and coronal, paramagnetic contrast-enhanced, fat saturated T1-weighted MRE sequence (**B**), showing impairment of the long segment of the distal ileum with stratification of the layers (arrows). Note also the fat proliferation adjacent to that segment, separating it from the other ileal loops.

Although they have their particularities, the imaging findings of inflammatory activity in Crohn’s disease observed by MRE are similar to those observed by CTE. In patients diagnosed with or suspected of having Crohn’s disease, various aspects should be assessed^13,24^: the thickness of the intestinal wall; the attenuation/signal intensity of the wall; the intensity and pattern of wall enhancement; the extent of involvement; stenosis or prestenotic dilatation (Figures 2 and 3); asymmetric lesions characteristic of Crohn’s disease; fistulas (Figures 3 and 4) and abscesses, which constitute the main complications of the disease; any engorgement of the *vasa recta*, known as the “comb sign” (Figure 5); fat proliferation, indicating chronicity of the inflammatory process (Figure 6); mesenteric adenopathy; mesenteric venous thrombosis; and any extraintestinal involvement of the disease.

When there is inflammatory activity, the wall thickening in either the small bowel or the colon is segmental, asymmetric, and discontinuous, associated with the wall stratification caused by mucosal hyperenhancement and submucosal edema. The normal thickness of the small bowel wall is typically 2-3 mm when its lumen is distended and considered abnormal when it exceeds 4-5 mm. When thickened, it can have a signal intensity/attenuation that is homogeneous or stratified, the latter characterized by alternation of the signal intensity/attenuation between the wall layers.

The lumen of the small bowel is considered distended when it exceeds 2.5 cm in diameter, which may occur due to the examination technique, since a large volume of fluid is introduced in a short space of time. Abrupt changes in caliber allow the identification of stenotic areas, which are characterized by wall thickening that causes a reduction in the intestinal lumen and upstream dilatation, the latter estimated to be more than 3 cm^13^. The diagnosis of stenosis is crucial since, in its presence, the use of capsule endoscopy should be avoided because of the increased risk of capsule retention, which is estimated to occur in up to 13% of patients with Crohn’s disease^25^.

The following signs indicate penetrating disease and mesenteric inflammation: fistulas, whether simple or complex-the latter may characterize the “star sign” (Figure 4); perianal fistulas that extend from the rectum/anus to the skin in the perineal region or vagina; abscesses and inflammatory masses-the latter poorly defined and heterogeneous with increased signal intensity/attenuation of the fatty or soft parts; the “comb sign”, indicating engorgement of the mesenteric vessels from the *vasa recta* to the inflamed loops (Figure 5); fat proliferation (or lipodystrophy), usually observed around the affected loops, characterized by fat hypertrophy that increases the spaces between the loops; mesenteric venous thrombosis, which can be acute (intraluminal thrombus) or chronic, when there is thinning of the central mesenteric veins with collateral circulation or varicose veins; and adenopathy, defined as lymph nodes with a short-axis diameter larger than 1.5 cm; lymph nodes with a short-axis diameter smaller than 1.5 cm are considered reactive and normal in Crohn’s disease^13^. Many of these findings are not specific to Crohn’s disease and can be seen in other diseases, such as infectious enteritis, ischemia, vasculitis, angioedema, mucositis, and graft-versus-host disease. Their specificity increases when they occur in the absence of other intra-abdominal inflammatory processes (e.g., tuberculosis, appendicitis, and diverticulitis) and when they coexist with asymmetry of wall changes and the penetrating complications typical of Crohn’s disease^13^.

In a study evaluating inflammation and fibrostenosis in Crohn’s disease^26^, CTE findings were shown to correlate well with the histopathology (Spearman’s r = 0.7 and 0.6 for inflammation and fibrostenosis, respectively; *p* < 0.0001 for both). In that study, the CTE variables found to be significantly associated with the pathological inflammation score were mucosal hyperenhancement (*p* = 0.04), wall thickening (*p* = 0.04), the “comb sign” (*p* < 0.0001), and lymph node enlargement (*p* = 0.016).

Some studies have also demonstrated the good accuracy of CTE and MRE in the assessment of Crohn’s disease complications such as stenosis, fistulas, and abscesses. In a study of 44 cases of Crohn’s disease^27^, CTE and MRE showed similar sensitivity and specificity for evaluating the inflammatory activity of Crohn’s disease (sensitivity of 85% and 92%, respectively; and specificity of 100% and 90%, respectively).

### Grading the inflammatory activity of Crohn’s disease

Although cross-sectional imaging methods show good efficacy in the diagnosis of patients with Crohn’s disease, the treatment and follow-up strategies in such patients are dictated by the severity of the inflammatory activity. A number of studies have sought to validate an index (or score) capable of representing the degree of inflammation of the disease to guide the therapeutic decisions^28,29^. There is as yet no universally accepted index. Ileocolonoscopy has been considered the gold standard to establish the severity of the disease and adopts a well-established index-the Crohn’s disease endoscopic index of severity-which considers the presence or absence of four endoscopic parameters (superficial ulcers, deep ulcers, ulcerated stenoses, and non-ulcerated stenoses), as well as the extent of and number of segments affected by the disease^30^. However, ileocolonoscopy has limitations, mainly because it is an invasive method, is limited to the assessment only as far as the distal ileum, and is unable to assess the extraluminal extent of the disease.

A recent meta-analysis evaluated the accuracy of the different methods of imaging (ultrasound, scintigraphy, MRE, and CTE) in assessing the degree of severity of Crohn’s disease. The authors showed that the accuracy of CTE (86%) and MRE (84%) was 86% and 84%, respectively, compared with 44% and 40%, respectively, for ultrasound and scintigraphy^28^. According to those authors, there is no widely accepted scoring system for grading the inflammatory activity of the disease. However, the Clermont score^31^ and the magnetic resonance index of activity^32^ have already been validated and have shown good accuracy in the detection of ulcers and of a treatment response in Crohn’s disease^33^.

Another recent study proposed an index that has shown a good correlation between MRE findings and histopathology in non-perforating small bowel Crohn’s disease^29^. The authors evaluated 16 patients who underwent MRE and small bowel resection, assessing the following parameters (Table 2): wall thickness; wall signal intensity, compared with that of the normal bowel, on T2-weighted images; perimural signal intensity on T2-weighted images; the “comb sign”; dimensions of the lymph nodes; and enhancement of the lymph nodes. The histopathology was found to correlate significantly with wall thickness (*p* < 0.001), wall signal intensity on T2-weighted images (*p* < 0.001), wall enhancement (*p* = 0.005), perimural signal intensity on T2-weighted images (*p* = 0.02), and the “comb sign” (*p* = 0.06), although not with the pattern of enhancement, dimensions of the lymph nodes, or enhancement of the lymph nodes.

**Table 2:** Suggested MRE parameters for the development of a qualitative index of Crohn's disease inflammatory activity.

Parameter	Score			
	0	1		3
Wall thickness	1-3 mm	> 3-5 mm	> 5-7 mm	> 7 mm
Wall signal intensity on T2-weighted images	Equivalent to that of a normal loop	Slight signal increase (dark gray)	Moderate signal increase (light gray)	Marked signal increase (white, similar to that of the luminal content)
Perimural signal intensity on T2-weighted images	Similar to normal mesentery	High, without fluid	Small (< 2 mm) fluid collection	Large (> 2 mm) fluid collection
Wall enhancement pattern	-	Homogeneous	Mucosal	Delaminated
Wall enhancement	Similar to that of the normal bowel	Sight increase (less than that of the vessels)	Moderate increase (but still less than that of the vessels)	Marked increase (similar to that of the vessels)
Lymph nodes	Absent	Clusters of lymph nodes < 1 cm	One lymph node > 1 cm	Three lymph nodes > 1 cm
Lymph node enhancement	Less than that of the vessels	Equivalent to or greater than that of the vessels	-	-
"Comb sign"	Absent	Present	-	-

One recent study evaluated the use of diffusion-weighted imaging in predicting the inflammatory activity of Crohn’s disease in the distal ileum, showing that it presents good accuracy in the detection of an inflammatory process, in comparison with the identification of inflammation by colonoscopy, with a sensitivity and specificity of 88.8% and 95.0%, respectively, when a cut-off point of 2.1 × 10^−3^ mm^2^/s for the ADC is applied^34^. Other studies have shown that diffusion-weighted imaging has the potential to substantially increase interobserver reproducibility in the evaluation of patients with Crohn’s disease^35^, making it a useful tool in the monitoring of treatment responses in such patients^36,37^, as well as a good predictor of capsule retention during capsule endoscopy^38^.

## CONCLUSION

The management and treatment of patients with Crohn’s disease require proper assessment of the extent and severity of the disease manifestations, as well as of the signs of acute inflammatory activity and possible complications.

In the evaluation and interpretation of Crohn’s disease in medical reports by radiologists, as well as in the choice between using MRE or CTE, physicians should be aware of certain aspects for which there is strong evidence^13^: Crohn’s disease is likely when there is hyperenhancement and wall thickening or when the inflammation is asymmetric and coexists with penetrating disease; the number of segments affected, the approximate location, and the extent and degree of the prestenotic dilatation should be reported; signs of wall inflammation should be reported when there is a penetrating disease (fistulas or abscesses); cross-sectional imaging methods should be used in the diagnosis of Crohn’s disease to identify penetrating disease and the segments that were inaccessible by endoscopic methods-in such cases, these methods should also be used in follow-up evaluations; MRE and CTE must always include the perianal region in the evaluation, although protocols for the evaluation of perianal disease, if available, should be followed; for the association with the severity of the endoscopic findings, a hyperintense wall signal on T2-weighted images, restricted diffusion, increased peri-intestinal fat density, wall thickening, and mural ulcers should be reported; and mesenteric venous thrombosis and varicose veins of the small bowel should always be evaluated.

Cross-sectional imaging methods are increasingly gaining ground, becoming essential in the diagnosis and follow-up of patients with Crohn’s disease, although their utility is limited in the initial stages of the disease, when the lesions are still subtle and limited to the mucosa. Currently, CTE is the cross-sectional method of choice in the evaluation of such patients at most health care facilities and is the method recommended by the American College of Radiology in most clinical situations related to Crohn’s disease^39^, although MRE has also shown very high accuracy. In addition, recent studies have demonstrated that MRE has become the reference standard in the assessment of the inflammatory activity of the disease, especially in perianal manifestations^40^.

Many studies have compared the effectiveness of MRE and CTE in the detection of active Crohn’s disease, suggesting that neither method is superior to the other. Most authors have reported that the two methods are similar in terms of image quality and effectiveness^41-43^. It is important to consider the advantages and limitations of each method, especially regarding the use of radiation in CTE and the greater susceptibility to artifacts in MRE. Therefore, in the long-term follow-up of patients with Crohn’s disease, MRE is preferable because it does not involve the use of ionizing radiation.

Knowledge of the main indications, contraindications, advantages, and disadvantages of MRE and CTE, as well as of the main imaging parameters to be evaluated in Crohn’s disease, should not be restricted to radiologists. The use of MRE and CTE is the present and future of the evaluation of the complex condition that is Crohn’s disease. Going forward, they will play ever greater roles and should be increasingly familiar to clinicians and surgeons.

## References

[1] Fiocchi C (1998). Inflammatory bowel disease: etiology and pathogenesis. Gastroenterology.

[2] Kim SH (2015). Computed tomography enterography and magnetic resonance enterography in the diagnosis of Crohn's disease. Intest Res.

[3] D'Incà R, Caccaro R (2014). Measuring disease activity in Crohn's disease: what is currently available to the clinician. Clin Exp Gastroenterol.

[4] Fletcher JG (2009). CT enterography technique: theme and variations. Abdom Imaging.

[5] D'Ippolito G, Braga FA, Resende MC (2012). Computed tomography enterography: a comparison of different neutral oral contrast agents. Radiol Bras.

[6] Macari M, Megibow AJ, Balthazar EJ (2007). A pattern approach to the abnormal small bowel: observations at MDCT and CT enterography. AJR Am J Roentgenol.

[7] Kambadakone AR, Prakash P, Hahn PF (2010). Low-dose CT examinations in Crohn's disease: impact on image quality, diagnostic performance, and radiation dose. AJR Am J Roentgenol.

[8] Lee SJ, Park SH, Kim AY (2011). A prospective comparison of standard-dose CT enterography and 50% reduced-dose CT enterography with and without noise reduction for evaluating Crohn disease. AJR Am J Roentgenol.

[9] Negaard A, Paulsen V, Sandvik L (2007). A prospective randomized comparison between two MRI studies of the small bowel in Crohn's disease, the oral contrast method and MR enteroclysis. Eur Radiol.

[10] Desmond AN, O'Regan K, Curran C (2008). Crohn's disease: factors associated with exposure to high levels of diagnostic radiation. Gut.

[11] Huang MP, Liang CH, Zhao ZJ (2011). Evaluation of image quality and radiation dose at prospective ECG-triggered axial 256-slice multi-detector CT in infants with congenital heart disease. Pediatr Radiol.

[12] Hammer MR, Podberesky DJ, Dillman JR (2013). Multidetector computed tomographic and magnetic resonance enterography in children: state of the art. Radiol Clin North Am.

[13] Bruining DH, Zimmermann EM, Loftus Jr EV (2018). Consensus recommendations for evaluation, interpretation, and utilization of computed tomography and magnetic resonance enterography in patients with small bowel Crohn's disease. Radiology.

[14] Schreyer AG, Geissler A, Albrich H (2004). Abdominal MRI after enteroclysis or with oral contrast in patients with suspected or proven Crohn's disease. Clin Gastroenterol Hepatol.

[15] Fidler JL, Guimaraes L, Einstein DM (2009). MR imaging of the small bowel. Radiographics.

[16] McDonald RJ, McDonald JS, Kallmes DF (2015). Intracranial gadolinium deposition after contrast-enhanced MR imaging. Radiology.

[17] Lanier MH, Shetty AS, Salter A (2018). Evaluation of noncontrast MR enterography for pediatric inflammatory bowel disease assessment. J Magn Reson Imaging.

[18] Froehlich JM, Waldherr C, Stoupis C (2010). MR motility imaging in Crohn's disease improves lesion detection compared with standard MR imaging. Eur Radiol.

[19] Fidler J (2007). MR imaging of the small bowel. Radiol Clin North Am.

[20] Sahni VA, Ahmad R, Burling D (2008). Which method is best for imaging of perianal fistula?. Abdom Imaging.

[21] Miguel Criado J, Salto LC, Rivas PF (2012). MR imaging evaluation of perianal fistulas: spectrum of imaging features. Radiographics.

[22] Burlin S, Favaro LR, Bretas EAS (2017). Using computed tomography enterography to evaluate patients with Crohn's disease: what impact does examiner experience have on the reproducibility of the method?. Radiol Bras.

[23] Silverberg MS, Satsangi J, Ahmad T (2005). Toward an integrated clinical, molecular and serological classification of inflammatory bowel disease: report of a Working Party of the 2005 Montreal World Congress of Gastroenterology. Can J Gastroenterol.

[24] Gore RM, Balthazar EJ, Ghahremani GG (1996). CT features of ulcerative colitis and Crohn's disease. AJR Am J Roentgenol.

[25] Fletcher JG, Fidler JL, Bruining DH (2011). New concepts in intestinal imaging for inflammatory bowel diseases. Gastroenterology.

[26] Chiorean MV, Sandrasegaran K, Saxena R (2007). Correlation of CT enteroclysis with surgical pathology in Crohn's disease. Am J Gastroenterol.

[27] Fiorino G, Bonifacio C, Peyrin-Biroulet L (2011). Prospective comparison of computed tomography enterography and magnetic resonance enterography for assessment of disease activity and complications in ileocolonic Crohn's disease. Inflamm Bowel Dis.

[28] Puylaert CAJ, Tielbeek JAW, Bipat S (2015). Grading of Crohn's disease activity using CT, MRI, US and scintigraphy: a meta-analysis. Eur Radiol.

[29] Steward MJ, Punwani S, Proctor I (2012). Non-perforating small bowel Crohn's disease assessed by MRI enterography: derivation and histopathological validation of an MR-based activity index. Eur J Radiol.

[30] Mary JY, Modigliani R (1989). Development and validation of an endoscopic index of the severity for Crohn's disease: a prospective multicentre study. Groupe d'Etudes Thérapeutiques des Affections Inflammatoires du Tube Digestif (GETAID). Gut.

[31] Buisson A, Joubert A, Montoriol PF (2013). Diffusion-weighted magnetic resonance imaging for detecting and assessing ileal inflammation in Crohn's disease. Aliment Pharmacol Ther.

[32] Rimola J, Ordás I, Rodriguez S (2011). Magnetic resonance imaging for evaluation of Crohn's disease: validation of parameters of severity and quantitative index of activity. Inflamm Bowel Dis.

[33] Buisson A, Pereira B, Goutte M (2017). Magnetic resonance index of activity (MaRIA) and Clermont score are highly and equally effective MRI indices in detecting mucosal healing in Crohn's disease. Dig Liver Dis.

[34] Durayski E, Watte G, Pacini GS (2019). Diffusion-weighted imaging and apparent diffusion coefficient values for evaluating terminal ileitis in patients with Crohn's disease. Radiol Bras.

[35] Kim JS, Jang HY, Park SH (2017). MR Enterography assessment of bowel inflammation severity in Crohn disease using the MR index of activity score: modifying roles of DWI and effects of contrast phases. AJR Am J Roentgenol.

[36] Huh J, Kim KJ, Park SH (2017). Diffusion-weighted MR enterography to monitor bowel inflammation after medical therapy in Crohn's disease: a prospective longitudinal study. Korean J Radiol.

[37] Klang E, Kopylov U, Eliakim R (2017). Diffusion-weighted imaging in quiescent Crohn's disease: correlation with inflammatory biomarkers and video capsule endoscopy. Clin Radiol.

[38] Klang E, Kopylov U, Ben-Horin S (2017). Assessment of patency capsule retention using MR diffusion-weighted imaging. Eur Radiol.

[39] Kim DH, Carucci LR, Baker ME (2015). ACR appropriateness criteria Crohn disease. J Am Coll Radiol.

[40] Vermeire S, Ferrante M, Rutgeerts P (2013). Recent advances: personalised use of current Crohn's disease therapeutic options. Gut.

[41] Lee SS, Kim AY, Yang SK (2009). Crohn disease of the small bowel: comparison of CT enterography, MR enterography, and small-bowel follow-through as diagnostic techniques. Radiology.

[42] Siddiki HA, Fidler JL, Fletcher JG (2009). Prospective comparison of state-of-the-art MR enterography and CT enterography in small-bowel Crohn's disease. AJR Am J Roentgenol.

[43] Jensen MD, Ormstrup T, Vagn-Hansen C (2011). Interobserver and intermodality agreement for detection of small bowel Crohn's disease with MR enterography and CT enterography. Inflamm Bowel Dis.

